# Enhancing the Photoresponsivity and External Quantum Efficiency of ReSe_2_ Photodetectors Through the Insertion of a Graphene Auxiliary Layer

**DOI:** 10.3390/s26010115

**Published:** 2025-12-24

**Authors:** Qiutong Liu, Beiyang Jin, Yutong Li, Peishuo Li, Jingyu Zhang, Yankun Chen, Chenkai Hu, Wei Li

**Affiliations:** School of Microelectronics, Northwestern Polytechnical University, 127 Youyi West Road, Xi’an 710072, China; liu_qiu_tong@mail.nwpu.edu.cn (Q.L.); king_alexander@mail.nwpu.edu.cn (B.J.); liyutong130329@mail.nwpu.edu.cn (Y.L.); lipeishuo@mail.nwpu.edu.cn (P.L.); zhangjingyu2@mail.nwpu.edu.cn (J.Z.); chenyankun@mail.nwpu.edu.cn (Y.C.); huchenkai@mail.nwpu.edu.cn (C.H.)

**Keywords:** 2D materials, ReSe_2_, photodetector, photoresponsivity

## Abstract

Two-dimensional (2D) materials demonstrate significant potential in photodetector technology. They offer high sensitivity, wide spectral range, flexibility and transparency, especially in infrared detection, promising advancements in wearable and flexible electronics. This study explores the application of 2D materials in high-performance photodetectors. Rhenium diselenide (ReSe_2_) was used as the channel, and graphene (Gr) was inserted between ReSe_2_ and SiO_2_ as the gate electrode to enhance device performance. A ReSe_2_/Gr heterostructure field-effect transistor (FET) was fabricated to investigate the role of Gr in improving the optoelectronic properties of ReSe_2_ phototransistors. Specifically, the ReSe_2_ FET without Gr auxiliary layer demonstrates a responsivity (R) of 294 mA/W, an external quantum efficiency (EQE) of 68.75%, and response times as brief as 40/62 ms. Compared with the ReSe_2_ phototransistor, the ReSe_2_/Gr phototransistor exhibits significantly improved photoresponsivity and EQE, with the photoresponsivity enhanced by a factor of ap-proximately 3.58 and the EQE enhanced by a factor of approximately 3.59. These enhancements are mainly attributed to optimization of interfacial band alignment and the strengthened photogating effect by Gr auxiliary layer. This research not only underscores the pivotal role of Gr in boosting the capabilities of 2D photodetectors but also offers a viable strategy for developing high-performance photodetectors with 2D materials.

## 1. Introduction

Nowadays, the photodetectors play a crucial role in thermal imaging, biomedical imaging, communication, military, and other significant fields. The photodetectors significantly influence various sectors today: silicon-based sensors in digital cameras, infrared units in 5G and thermal imaging, and ultraviolet detectors in disaster prevention and fire alarms [1,2,3,4]. Hence, in the intelligent information era, it is essential to advance materials and processes for high-performance photodetectors. While the traditional photosensitive materials like Si, Ge, InGaAs, and HgCdTe are established, their limitations in high-performance photodetection are becoming apparent. The Si-based detectors are limited in near-infrared and longer wavelengths due to bandgap constraints [5]. The devices using epitaxially grown InGaAs and HgCdTe, although effective in infrared ranges, are expensive and require complex fabrication and low-temperature operation [6]. As Moore’s law approaches its limits, the challenges in miniaturization and performance degradation of these materials are impeding future progress in photodetection research [7]. Due to these drawbacks, there has been an urgent need in recent years to break the limitations of traditional materials and find new photosensitive materials to provide the low-cost and high-performance photodetector fabrication solutions. Recently, many impressive achievements of two-dimensional (2D) material-based photodetectors have been reported, including ultrahigh photoresponsivity [8], ultrafast photo response speed [9], ultrabroad detecting band [10], and ultrasensitive photodetection [11] due to their unique electronic and optoelectronic properties.

The family of 2D transition metal dichalcogenides (TMDCs) is expansive and has gained prominence in photodetector research due to their adjustable bandgaps and superior optical absorption. The WSe_2_/SnSe_2_ heterostructure photodetector can achieve band alignment modulation from Type II to Type III through the regulation of gate and drain voltages [12]. Thanks to the sharp changes in the tunneling current, this device achieved an ultra-short response time of approximately 1 μs and a negative photoresponsivity up to the order of 10^4^ A/W [12]. The researchers apply the energy band engineering of 2D material van der Waals heterostructures to fabricate a WSe_2_/MoS_2_ heterojunction p-n junction, which exhibited high photoresponsivity of 2700 A/W, a specific detectivity of 5 × 10^11^ Jones, and an excellent photoelectric performance with a response time of 17 ms [13]. Among TMDCs, ReSe_2_ has attracted significant interest due to its unusual 1T′ structure and extraordinary properties in various fields over the past years [14]. The ReSe_2_ possesses large bandgaps (1.3 eV), distinctive interlayer decoupling, and strong anisotropic properties, which endow more degree of freedom for constructing novel optoelectronic devices [15]. An ReSe_2_ photodetector encapsulated in h-BN with Sc/Au electrodes achieved a mobility of 166 cm^2^ V^−1^ s^−1^ and an ultrahigh responsivity of 3.2 × 10^6^ A/W, thanks to its low Schottky barrier and low-resistance contacts [16]. This device also demonstrated a near-infrared spectral response through doping with Co [16]. In addition, Co doping broadens the photoresponse of the device into the near-infrared region, which is attributed to the additional trap states introduced by Co dopants within the bandgap of ReSe_2_. A photodetector based on a WS_2_/ReSe_2_ vdW heterostructure demonstrates an extraordinary photoresponse of 2.78 mA/W and a specific detectivity of 1.05 × 10^10^ Jones under 350 nm illumination in self-powered mode [17]. Additionally, its response speed significantly surpasses [9] that of most currently published self-driven photodetectors [17]. Furthermore, the scientists have also developed a novel polarization photodetector based on a ReSe_2_/PdSe_2_ heterostructure. This device offers a broadband response from near-ultraviolet to near-infrared with a responsivity of up to 313 mA/W and fast photoswitching speeds, with rise and fall times of about 70 and 82 μs [18]. These results highlight the significant potential of the ReSe_2_/PdSe_2_ heterostructure for high-performance polarization detection [18]. The researchers also designed a ReS_2_/ReSe_2_ heterojunction phototransistor with thin hafnium oxide as a localized back-gate dielectric, exhibiting a rectification ratio of approximately 10^3^ and significant gate tunability with an off-state current of 433 fA and an on/off current ratio exceeding 10^6^ [19]. This device responded to broadband excitation from 400 to 633 nm with a photoresponsivity of about 10^4^ A/W [19]. The significant progress has been achieved in enhancing the photosensitivity and response speed of ReSe_2_ photodetectors, with potential for further improvements through advanced techniques. However, the low quantum efficiency, high noise, and slow response speed caused by the thinness of 2D materials limit their application in photodetectors [20]. Specifically, the photoelectric detectors with the photoconductive type are suffering from the low external quantum efficiency and slow response speed due to the thinness of two-dimensional materials leading to higher resistance and increased impact of defects [20]. Therefore, the improvements are necessary for photodetectors based on monolayer ReSe_2_. A common method in the scientific community involves chemically doping the photosensitive semiconductor material to adjust the sensitivity and spectral response range of the photodetectors [21,22,23]. Additionally, the techniques such as quantum well structures and heterojunction configurations can be employed to enhance photodetector performance [24,25]. However, the performance enhancements of photodetectors based on chemical doping complicate the fabrication process, increase manufacturing costs, and reduce controllability [26]. To address these challenges, this work focuses on the ReSe_2_/graphene (Gr) heterostructure, where ReSe_2_ was used as the channel, and Gr was inserted between ReSe_2_ and SiO_2_ as the gate electrode to enhance device performance. Graphene’s role in the ReSe_2_/Gr heterojunction is twofold: (1) forming a built-in electric field with ReSe_2_ to facilitate the efficient separation of photogenerated electron-hole pairs; (2) trapping photogenerated holes via surface defects to suppress carrier recombination, induce a local positive photogating voltage, and enhance the electron concentration in the ReSe_2_ channel. In contrast, in related studies, graphene is mostly used for circuit connections: either leveraging its ohmic contact with gold (Au) to reduce electrode transmission loss and boost photocurrent [27]; or serving as a bottom electrode inserted between the device and the silicon dioxide (SiO_2_) substrate [28]. In summary, this study differs substantially from other ReSe_2_/Gr device research in terms of structure and mechanism.

The graphene (Gr), representing 2D materials, with its atomic thickness, smooth surfaces without dangling bonds, and absence of surface states, shows tremendous potential in photodetection and hetero integration [29,30]. In this study, we prepared Gr-assisted ReSe_2_ phototransistors, characterized the channel materials used in fabrication, and tested the electrical and optical properties of the produced phototransistors. Additionally, we researched and analyzed the mechanism underlying the performance of the Gr-assisted ReSe_2_ phototransistors. Under the control of back-gate voltage, the device achieved an ultra-low dark current of 1.39 × 10^−12^ A and an extremely high on/off ratio of 3.9 × 10^6^, with an electron mobility of 8.5 cm^2^ V^−1^ s^−1^ extracted for the ReSe_2_/Gr channel. The introduction of Gr enhances the photogating effect, resulting in a ReSe_2_ phototransistor with the Gr auxiliary layer achieving a high photoresponsivity of 294 mA/W, a specific detectivity of 1.74 × 10^11^ Jones, an external quantum efficiency of 68.75%, and a fast response time of 40/62 ms. Compared to ordinary ReSe_2_ phototransistors, the Gr auxiliary layer improves the photoresponsivity by approximately 258.5% and the external quantum efficiency by about 259.4%. Finally, we analyzed the working mechanism of the device with the help of energy band and clarified the main reasons for the Gr auxiliary layer to improve the photoresponsivity. We have also conducted a comparative analysis with optoelectronic devices reported recently, confirming that the performance of the device in this study has achieved significant improvement [31,32,33,34,35].

## 2. Materials and Methods

### 2.1. Device Fabrication

In this research, the ReSe_2_ and Gr flakes were derived through mechanical exfoliation. The ReSe_2_ and Gr crystals were bought from Six Carbon Technology Shenzhen (Shenzhen, China). The process involved placing large ReSe_2_ (Gr) pieces on tape, which were then folded, adhered, and peeled multiple times to extract thin nanosheets with poly (dimethylsiloxane) (PDMS). These Gr flakes, once achieving the desired thickness, along with similarly procured ReSe_2_ flakes, were transferred to a 285 nm SiO_2_/Si substrate using an accurate transfer system (Metatest, E1-T, Nanjing, China). The Au electrodes with the thickness of 50 nm were precisely created by standard UV lithography and thermal evaporation in advance, and then the Au electrodes were transferred on both side of ReSe_2_ channel to form the source and drain electrodes. The gold source materials used to evaporate electrodes were purchased from Beijing Dream Material Technology Co., Ltd. (Beijing, China). The final step involved annealing the device in a vacuum at 200 °C for 120 min to remove resisted residues and to improve electrical contact.

### 2.2. Characterization and Measurement

The dimensions and structural properties of both Gr and ReSe_2_ flakes were assessed using optical microscopy, atomic force microscopy (AFM, Bruker, Billerica, MI, USA), and Raman spectroscopy (Horiba Jobin Yvon, Kyoto, Japan, 532 nm excitation laser). The devices underwent electrical evaluation with a Keysight B2912A source meter (Keysight Technologies, Santa Rosa, CA, USA) and photoelectric testing using a 532 nm laser and a probe station setup. These comprehensive assessments were carried out under normal atmospheric conditions, in air, at room temperature.

## 3. Results and Discussion

**Figure 1:**
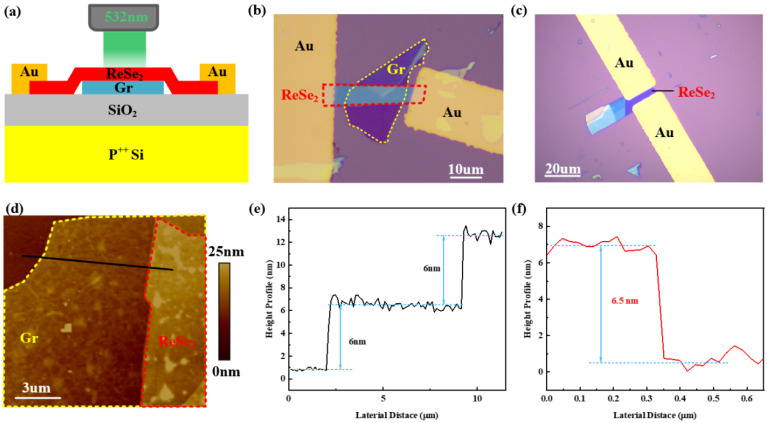
The schematic of ReSe_2_/Gr and ReSe_2_ FETs as well as AFM characterizations. (**a**) Schematic of ReSe_2_/Gr heterojunction FET. (**b**) Optical microscopy image of ReSe_2_/Gr heterojunction FET. Gr and ReSe_2_ are marked with yellow and red dot lines, respectively. (**c**) Optical microscopy image of ReSe_2_ FET. (**d**) The AFM image of ReSe_2_/Gr heterojunction FET, with Gr and ReSe_2_ outlined in yellow and red dot lines, respectively. (**e**) AFM height profiles of the ReSe_2_/Gr heterojunction FET. The first step of the curve indicates the thickness of Gr flake, and the second step indicates the thickness of ReSe_2_ flake. (**f**) AFM height profiles of the ReS_2_ flake in ReSe_2_ FET.

Figure 1a illustrates the schematic of the ReSe_2_/Gr heterojunction field-effect transistor (FET) on a 285 nm SiO_2_/Si substrate. The heterostructure is constructed by vertically stacking ReSe_2_ and Gr flakes to form the photosensitive channel of the phototransistors, with gold (Au) serving as the source/drain electrodes. The design strategically exposes the heterojunction to incident light by extending electrodes from the sides, where the electrodes are in direct contact with ReSe_2_ flakes but without Gr. The optical microscopy images of ReSe_2_/Gr heterojunction FET are presented in Figure 1b. In Figure 1b, Gr and ReSe_2_ are marked with yellow and red dotted lines, respectively, with their overlapping area being 19 μm in length and 6 μm in width. To assess the impacts of Gr under the ReSe_2_ channel on the electrical and photoelectrical properties of ReSe_2_/Gr heterojunction FET, we also constructed ReSe_2_ back-gate transistors without a Gr layer under identical conditions for comprehensive characterizations and measurements. The schematic of ReSe_2_ back-gate transistors is depicted in Figure S1a (Supporting Information). The optical microscopy image of ReSe_2_ back-gate transistor is presented in Figure 1c. As shown in Figure 1b,c, the only difference between the two types of devices lies in whether Gr is inserted or not—the source and drain electrodes are both in direct contact with ReSe_2_, and Gr is not connected to the electrodes. Therefore, the Schottky barrier and contact resistance of the two devices show little difference, which will not exert a significant impact on the performance comparison between them. Both devices were prepared using mechanical exfoliation and dry transfer method. The employed dry transfer method ensures a smooth and uncontaminated surface for both the channel material and the van der Waals electrodes, maintaining cleanliness around the channel area. The ReSe_2_/Gr and ReSe_2_ FETs underwent initial characterization to determine material thickness and surface morphology using an atomic force microscope (AFM). The AFM image in Figure 1d highlights the heterojunction area of ReSe_2_/Gr FET, with Gr and ReSe_2_ outlined in yellow and red dotted lines, respectively. This image demonstrates a smooth channel surface and negligible interlayer bubbles resulting from the dry transfer technique within the heterojunction region. The extracted height data along the black solid line in Figure 1d produce the height curve shown in Figure 1e. The first step of the curve indicates the thickness of Gr is about 6 nm, and the second step indicates the thickness of ReSe_2_ is also 6 nm, revealing that the thickness of each layer in ReSe_2_/Gr heterojunction is approximately 6 nm. Furthermore, Figure 1f confirms that the thickness of ReSe_2_ flake in the ReSe_2_ FET is about 6.5 nm, as evidenced by its AFM image in Figure S1b (Supporting Information). The consistent thickness can eliminate the influences of flake thickness on device performance during later devices characteristics comparisons.

**Figure 2:**
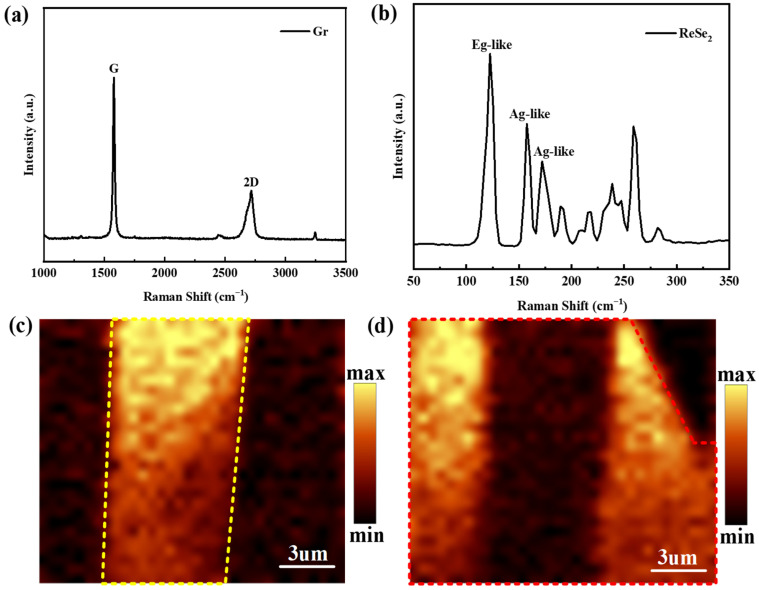
The Raman characterizations of ReSe_2_/Gr FET. (**a**) The Raman spectrum of Gr in ReSe_2_/Gr FET. (**b**) The Raman spectrum of ReSe_2_ in ReSe_2_/Gr FET. (**c**) The Raman mapping image at 123 cm^−1^ in ReSe_2_/Gr FET. (the bright yellow region with yellow dashed outlines corresponds to the ReSe_2_ area, highlighting its higher-intensity E_1g_ peak); (**d**) The Raman mapping image at 1580 cm^−1^ in ReSe_2_/Gr FET. (The bright yellow region with red dashed outlines corresponds to the Gr area, indicating its pronounced G peak).

Furthermore, Raman spectroscopy, an optical non-destructive technique, was employed to analyze ReSe_2_ and Gr nanosheets to assess the quality of these 2D materials. To prevent laser-induced damage to the materials, a laser power of 0.5 mW and a spot diameter of approximately 300 nm were employed, focusing on the materials through a 100× objective lens. The signal-to-noise ratio of the Raman signals was enhanced through multiple integrations, with three repetitions and an integration time of 20 s each. The Raman spectrum, depicted in Figure 2a, highlights the G and 2D peaks of Gr nanosheets at 1580 cm^−1^ and 2717 cm^−1^, respectively, indicating sharp G peaks for in-plane vibrations of sp^2^ hybridized carbon atoms and 2D peaks for double phonon resonance, reflecting interlayer carbon atom stacking [36]. The absence of a significant D peak suggests minimal structural defects in the mechanically exfoliated Gr, indicative of high quality [36,37]. Notably, the G peak intensity surpasses the 2D peak in multilayer Gr, aligning with AFM-derived thickness data. Figure 2b presents the Raman spectrum of ReSe_2_ nanosheets, characterized by multiple sharp peaks due to its complex crystal structure and in-plane anisotropy, enhanced by additional valence electrons from Re atoms that contribute to intricate lattice vibrations. The complexity of the spectrum exceeds that of other 2D materials, with ReSe_2_ exhibiting 18 Raman-active modes (A_g_), 15 infrared-active modes (A_u_), and 3 acoustic modes within the 100–300 cm^−1^ range [31,32]. The presence of a dominant vibrational peak at 123 cm^−1^, attributed to the in-plane E_1g_ mode, and additional peaks at 159 cm^−1^ and 173 cm^−1^, associated with the A_1g_ mode, corroborates with identification in other studies for ReSe_2_ material [38,39,40]. In contrast to Gr, the thickness estimation for ReSe_2_ via Raman spectroscopy is challenged due to the weak interlayer coupling from Peierls distortion within its distorted 1T-phase lattice structure [40].

We also utilized Raman mapping mode to examine the ReSe_2_/Gr heterojunction device, resulting in the Raman mapping images depicted in Figure 2c,d. Figure 2c illustrates the Raman mapping at 123 cm^−1^, highlighting the higher intensity E_1g_ peak of the ReSe_2_ region, showcased in bright yellow with yellow dashed outlines. Conversely, Figure 2d reveals the Raman mapping at 1580 cm^−1^, indicating a pronounced G peak of the Gr region, illustrated in bright yellow with red dashed outlines. Notably, the Raman intensity in the overlapping region of Gr and ReSe_2_ at 1580 cm^−1^ is considerably reduced compared to the exposed Gr regions. This reduction is attributed to the impacts of the heterostructure, with ReSe_2_ absorbing part of the 532 nm incident light, diminishing the laser intensity on the underlying Gr. Additionally, the upper layer of ReSe_2_ partially absorbs the Raman scattering signal from Gr, affecting its return to the spectrometer.

**Figure 3:**
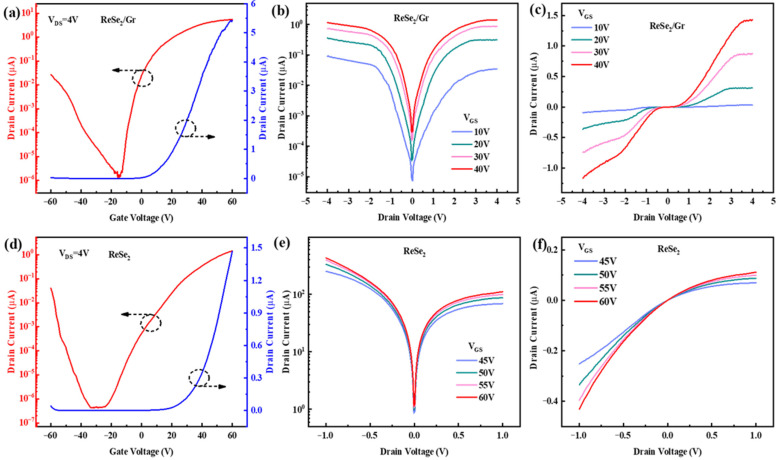
The electrical characteristics of ReSe_2_/Gr and ReSe_2_ FETs under the dark condition. (**a**) The transfer characteristic curves of the ReSe_2_/Gr phototransistor under the dark condition. (**b**) The semi-logarithmic output characteristic curves of the ReSe_2_/Gr phototransistor under the different gate voltages. (**c**) The linear output characteristic curves of the ReSe_2_/Gr phototransistor. (**d**) The transfer characteristic curves of the ReSe_2_ phototransistor under the dark condition. (**e**) The semi-logarithmic output characteristic curves of the ReSe_2_ phototransistor under the different gate voltages. (**f**) The linear output characteristic curves of the ReSe_2_ phototransistor.

Before testing the photoelectrical performance of the ReSe_2_/Gr heterojunction phototransistor, their electrical characteristics were first analyzed under the dark condition. The transfer characteristic curves of the ReSe_2_/Gr phototransistor, as shown in Figure 3a, sweep the gate-source voltage (V_GS_) from −60 V to +60 V with a fixed drain-source voltage (V_DS_) of 4 V. The curve shown in red on a semi-logarithmic scale exhibits the bipolar behavior of the device. Within −60 V < V_GS_ < −15 V, the drain current gradually decreases from 2.65 × 10^−8^ A to 1.39 × 10^−12^ A, exhibiting weak p-type behavior; when −15 V < V_GS_ < 60 V, as the gate voltage increases, the drain current gradually increases from 1.39 × 10^−12^ A to 5.42 × 10^−6^ A, with the current on/off ratio of the device reaching up to 3.9 × 10^6^, displaying significant n-type characteristics. The blue curves, representing the linear scale, predominantly display n-type behavior. Furthermore, these curves also confirms the effective modulation of the ReSe_2_ channel by the back gate voltage, with minimal impact from the shielding effect of Gr.

Additionally, we calculated the mobility of the device based on the transfer curve. The capacitance of SiO_2_ ($C_{SiO_{2}}$) was calculated using Equation (1), where *ε*_0_, *ε*_SiO2_ and *t*_SiO2_ denotes the permittivity of vacuum (8.85 × 10^12^ F/m), relative permittivity of SiO_2_ (3.9), thickness of SiO_2_ (285 nm), respectively. The calculated value of $C_{SiO_{2}}$ is 1.21 × 10^−8^ F/cm^2^. C_Gr_ was calculated using the same method, with a value of 6.64 × 10^−7^ F/cm^2^.

(1) $$C_{SiO_{2}}=\varepsilon_{0}\varepsilon_{SiO_{2}}/t_{SiO_{2}}$$

Given that Gr is situated at the bottom of ReSe_2_, the gate capacitance is constituted by a series arrangement of 285 nm SiO_2_ and 6 nm Gr. Consequently, the overall gate capacitance (C_g_) is calculated using Formula (2).

(2) $$\frac{1}{C_{g}}=\frac{1}{C_{SiO_{2}}}+\frac{1}{C_{Gr}}$$

The overall C_g_ was determined to be 1.19 × 10^−8^ F/cm^2^. Additionally, the linear fit result from Figure 3a with a slope of 1.27138 × 10^−7^ further facilitated the calculation of mobility *μ* = 8.5 cm^2^ V^−1^ s^−1^, according to Formula (3). Herein, L, W, and C_ox,_ respectively, denote the channel length, channel width, and gate dielectric capacitance, with the channel length being 19 μm and the channel width being 6 μm in the calculations.

(3) $$\mu =\frac{\Delta I_{DS}}{\Delta V_{GS}}\frac{L}{WC_{ox}V_{DS}}$$

The output characteristic curves were obtained by varying the V_DS_ from −4 V to 4 V under V_GS_ of 10 V, 20 V, 30 V, and 40 V, as depicted in Figure 3b,c. The semi-logarithmic output curves in Figure 3b indicate consistent metal-semiconductor contacts between the source/drain electrodes and the ReSe_2_ channel, showcasing the stability and symmetry of the output curves, hence reflecting the reliability of the transfer electrode process. Unlike conventional heterojunction devices, the involvement of Gr does not introduce the typical rectifying behavior, as both electrodes are in contact with the same material (ReSe_2_). The linear output curve in Figure 3c suggests a nonlinear correlation between the drain current and V_DS_ in the ReSe_2_/Gr phototransistors. Particularly noted at V_GS_ = 40 V, when 0 V < V_DS_ < 0.5 V, the drain current remains almost unchanged with an increase in V_DS_; when V_DS_ > 0.5 V, as V_DS_ increases, the drain current also increases without showing saturation within the tested range. This non-linearity can be attributed to Schottky contacts between the electrodes and the ReSe_2_ channel, causing significant voltage drops across these contacts under current flow.

Next, we conducted tests on ReSe_2_ transistor under dark conditions, shown in Figure 3d–f, revealing distinct electrical behaviors. Figure 3d displays the transfer curves under V_DS_ = 4 V, where the drain current reduces from 4.08 × 10^−8^ A to 4.22 × 10^−13^ A within −60 V < V_GS_ < −34 V, showing weak p-type behavior; when −25 V < V_GS_ < 60 V, as the gate voltage increases, the drain current gradually increases from 4.57 × 10^−13^ A to 1.39 × 10^−6^ A, indicating n-type behavior. The device demonstrates an on-state current of 1.39 μA (V_GS_ = 60 V, V_DS_ = 4 V) and an off-state current of 4.57 × 10^−7^ A (V_GS_ = −25 V, V_DS_ = 4 V), achieving an on/off ratio of nearly 3 × 10^6^. By utilizing Formula (3), the mobility was also calculated to be 0.29 cm^2^ V^−1^ s^−1^. Figure 3e depicts the semi-logarithmic output characteristic curves under the dark conditions when V_GS_ increases from 45 V to 60 V. Figure 3f corresponds to the output curve under linear coordinates, showing that as V_DS_ increases, I_DS_ also increases, indicating that the dry transfer process between the electrode and ReSe_2_ nanosheets has promoted effective Ohmic contact, thereby enhancing the contact characteristics. The tested electrical characteristic results of ReSe_2_/Gr heterojunction and ReSe_2_ transistors fully demonstrate that the Gr auxiliary layer does not seriously affect the performance of the device.

**Figure 4:**
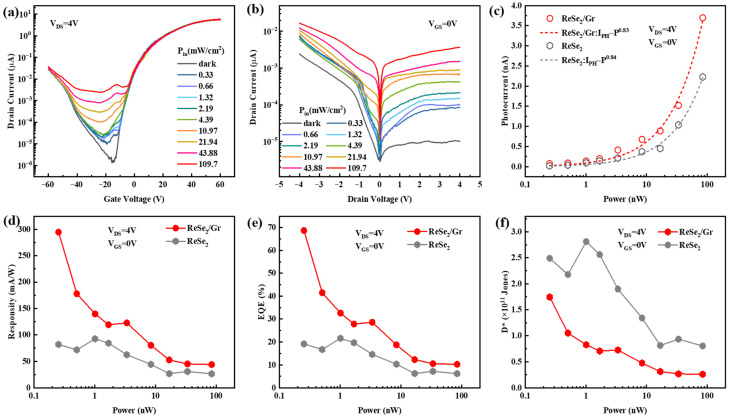
The photodetection performance of ReSe_2_/Gr and ReSe_2_ devices. (**a**) The transfer characteristic curves of the ReSe_2_/Gr phototransistor under different illumination intensities. (**b**) The semi-logarithmic output characteristic curves of the ReSe_2_/Gr phototransistor under different illumination intensities. (**c**) The relationship between net photocurrent and incident light power of the two devices. (**d**) The responsivity of the two devices from 0.25 nW to 84.10 nW of incident light power at operating conditions of V_DS_ = 4 V, V_GS_ = 0 V. (**e**) The external quantum efficiency of the two devices from 0.25 nW to 84.10 nW of incident light power at operating conditions of V_DS_ = 4 V, V_GS_ = 0 V. (**f**) The specific detectivity of the two devices from 0.25 nW to 84.10 nW of incident light power at operating conditions of V_DS_ = 4 V, V_GS_ = 0 V.

Because photodetection performance is an important indicator of photodetectors, we employed a power-adjustable 532 nm laser as the incident light source to assess the photodetection capabilities of the ReSe_2_/Gr heterojunction and ReSe_2_ phototransistors. As shown in Figure S2, the absorption of 532 nm light by ReSe_2_ is at a relatively balanced level—the absorption coefficient is neither excessively high nor too low. Therefore, we selected 532 nm, the wavelength at which ReSe_2_ exhibits balanced absorption to conduct the experiment and ensure its reliability. To facilitate observation of the gate voltage modulation effect on ReSe_2_/Gr devices, we characterized the photodetection performance by measuring the transfer characteristics curves under varying light intensities at V_DS_ = 4 V. Figure 4a illustrates these curves under both dark and illuminated conditions, with V_GS_ ranging from −60 V to +60 V and incident light power density from 0.33 mW/cm^2^ to 109.7 mW/cm^2^, with a spot diameter of 30 μm. The dark gray curve in Figure 4a represents the transfer characteristics without illumination, while the other curves depict its behavior under different illumination conditions. Clearly, under illumination, the output current of the device significantly increases, correlating with the incident light intensity. Notably, the transfer characteristic curves shift upward and to the left under illumination, especially with reference to the small valleys appearing around V_GS_ of −20 V to −10 V. This shift suggests that the photodetection performance primarily stems from the photoconductive effect, represented by the upward shift, and the photogating effect, indicated by the leftward shift. Since the leftward shift is small, the photogating effect needs to be further determined by fitting the net photocurrent with the incident light power using the formula I_PH_∝P^α^, where the I_PH_ is net photocurrent, calculated by subtracting the drain current under dark conditions (I_dark_) from the drain current under illumination (I_light_). Figure 4b depicts the output curves with different power density intensities, showing that output current increases with the rising of light power. Additionally, the transfer and output curves of the ReSe_2_ device are shown in Figure S3a,b (Supporting Information).

Subsequently, several important photodetection parameters were calculated based on the transfer and output curves. As shown in Figure 4c by the red circular symbols, we extracted the net photocurrent (I_PH_) of the device from 0.25 nW to 84.10 nW of incident light power (P) at V_DS_ = 4 V, where incident light power is defined as the laser power irradiated on the device, equivalent to the product of incident light power density (P_in_) and the channel area of the device (S) using formula *P* = *P*_in_ × *S*. It can be seen that with the increase in incident light power, the number of photo-generated carriers in the channel increases, and the net photocurrent of the device also increases. To further explore the mechanism of photocurrent generation, we fitted the relationship between net photocurrent and incident light power using the formula I_PH_ ∝ P^α^, where the fitting coefficient α equals 1 representing that the photocurrent is entirely from the photoconductive effect. The red dashed line in Figure 4c shows the fitting result α = 0.83 for the ReSe_2_/Gr device, and the gray dashed line shows the fitting result α = 0.84 for the ReSe_2_ device, indicating that the photocurrent source of both the ReSe_2_/Gr and ReSe_2_ devices is not solely from the photoconductive effect, but also from a combination of photoconductive and photogating effects due to the capture of photo-generated carriers by traps existing in the photosensitive channel and the interface between ReSe_2_ and Gr.

(4) $$EQE=\frac{hc}{q}\frac{R}{\lambda }$$

(5) $$D^{*}=R\sqrt{\frac{S}{2qI_{dark}}}$$

According to the formula *R* = *I*_PH_/*P*_in_ × *S*, we further calculated the responsivity (R). Figure 4d shows the variation in responsivity of the device from 0.25 nW to 84.10 nW of incident light power at the operating conditions of V_DS_ = 4 V and V_GS_ = 0 V. The responsivity is observed to decrease gradually with increasing incident light power. The ReSe_2_/Gr device reaches a maximum responsivity of 294 mA/W at an incident light power of 0.25 nW, marking a 258.5% increase over the ReSe_2_ device with a maximum responsivity of 82 mA/W. Under the low incident light power conditions, the device demonstrates enhanced responsivity. However, with an increase in incident light power, the concentration of photo-generated electron-hole pairs rises, which elevates the likelihood of carrier recombination and consequently diminishes the responsivity of the device. The external quantum efficiency (EQE) and specific detectivity (D*) are two other key performance parameters of the photodetector. The EQE reflects the efficiency of the photodetector in converting photons into electrons under real-world operating conditions, calculated as the ratio of the number of electrons collected by the photodetector to the number of incident photons. The EQE and D* can be calculated by Formulas (4) and (5), where h represents Planck’s constant (6.626 × 10^−34^ Js), c is the speed of light in a vacuum (3 × 10^8^ m/s), q is the elementary charge (1.602 × 10^−19^ C), and λ denotes the wavelength of the incident light. As shown in Figure 4e,f, the variations in EQE and D* of the two devices were calculated using Formulas (3) and (4) under operating conditions of V_DS_ = 4 V and V_GS_ = 0 V, ranging from 0.25 nW to 84.10 nW of incident light power, respectively. Similarly to the trend of responsivity with increasing incident light power, the EQE and D* gradually decrease with increasing incident light power. For the ReSe_2_/Gr device, the EQE is calculated to be 68.75% under a optical power of 0.25 nW, with a maximum detectivity (D*) of 1.74 × 10^11^ Jones. Compared to the pristine ReSe_2_ device (EQE = 19.15%), the EQE of the ReSe_2_/Gr heterostructure device is enhanced by a factor of 3.59. However, the D* of ReSe_2_/Gr device is less than that of ReSe_2_ device. From Figure 4b and Figure S3b (Supporting Information), we obtain that under the dark conditions of V_GS_ = 0 V and V_DS_ = 4 V, the I_dark_ for ReSe_2_/Gr and ReSe_2_ devices are 1.0 × 10^−5^ μA and 2.6 × 10^−7^ μA, respectively. Referring to Formula (5), since the dark current of ReSe_2_/Gr device is much greater than that of ReSe_2_ devices, the D* of ReSe_2_/Gr device under the same conditions is less than that of ReSe_2_ device.

**Figure 5:**
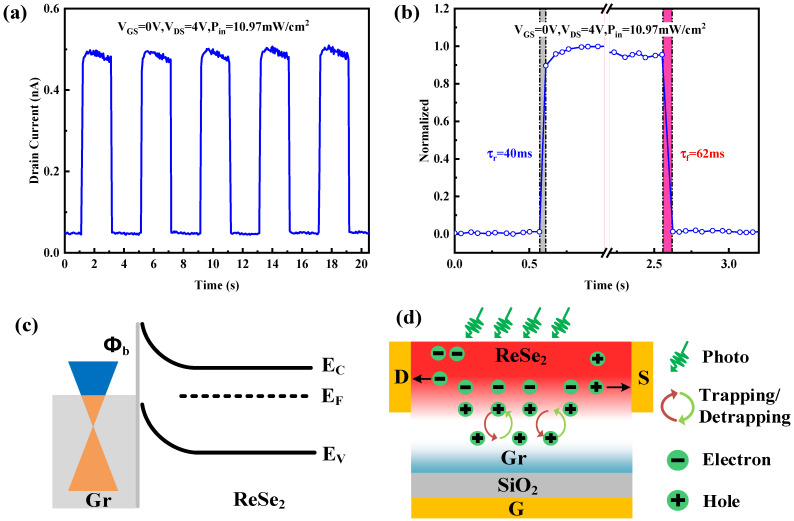
(**a**) The response of ReSe_2_/Gr phototransistor to light pulse. (**b**) The normalization of a single light pulse cycle showing the rise and fall times. (**c**) The energy band of ReSe_2_/Gr heterojunction under the condition of thermal equilibrium. (**d**) The transport mechanism of photogenerated carriers in ReSe_2_/Gr heterojunction FET.

Finally, we explored the light pulse output characteristics of ReSe_2_/Gr and ReSe_2_ phototransistors under operational conditions of V_DS_ = 4 V and V_GS_ = 0 V, highlighting the swift photoresponse capabilities as a photodetector. By employing a signal generator to meticulously control an adjustable light source, we produced laser pulses with durations and intervals of 2 s and 2 s, respectively. Figure 5a illustrates the response of ReSe_2_/Gr phototransistor to light pulse with the power density of 10.97 mW/cm^2^, showing a rapid increase in drain current upon light exposure and a swift decrease when the light ceases. Remarkably, the ReSe_2_/Gr phototransistor maintains its performance after several pulse tests, underscoring its exceptional stability and repeatability. The light pulse response parameters such as rise time (*τ*_r_) and fall time (*τ*_f_), crucial for assessing response speeds of photodetectors, were also analyzed. Specifically, the *τ*_r_ is defined as the time required for the output current value to rise from 10% of the peak value to 90% of the peak value; *τ*_f_ is defined as the time required for the output current value to drop from 90% to 10% of the peak value. Further normalization of a single light pulse cycle as shown in Figure 5a is depicted in Figure 5b, where we observed a rise time of 40 ms and a fall time of 62 ms, demonstrating the quick response of ReSe_2_/Gr phototransistor. In Figure S4a (Supporting Information), we also measured the light pulse output characteristics of the ReSe_2_ device under the same conditions and the normalized single light pulse is shown in Figure S4b (Supporting Information). The extracted rise time from Figure S4b (Supporting Information) is 50 ms, and the fall time is 67 ms. The similar level of optical response time also fully proves that the Gr auxiliary layer does not affect the optical response speed of the device [41,42,43,44].

We also compared the key performance parameters of the ReSe_2_/Gr phototransistor with those of the ReSe_2_ phototransistor, as shown in Table 1. The ReSe_2_/Gr phototransistor exhibits higher photoresponsivity and EQE, with the photoresponsivity enhanced by a factor of approximately 3.58 and the EQE enhanced by a factor of approximately 3.59. The light response speeds of both the ReSe_2_/Gr and conventional ReSe_2_ phototransistors reach the sampling limit of the measurement equipment constrained by the sampling rate. Notably, the ReSe_2_/Gr device features a channel length of 19 μm, in contrast to the 4.1 μm channel of the ReSe_2_ device. Although the ReSe_2_/Gr device has a larger light-receiving area, the longer ReSe_2_ channel between the source and drain leads to a higher recombination probability of the transported photogenerated carriers. The actual number of photogenerated carriers contributing to the photocurrent in these two devices should be roughly equivalent, making the performance comparison between them fair.

To further analyze the working mechanism of the device and clarify why the Gr auxiliary layer improves responsivity, we elucidate the working mechanism of the ReSe_2_/Gr phototransistor using energy band theory. According to previous studies, the bandgap width (E_g_) of ReSe_2_ is 1.14 eV, and its electron affinity is 3.67 eV [27]. Graphene, a semimetal, presents a distinct band structure characterized by Dirac cones at the Fermi level, diverging significantly from typical semiconductors. This arrangement affords Gr extraordinary properties, including high carrier mobility and the anomalous quantum Hall effect. Notably, the work function of Gr ranges from 4.30 ± 0.05 eV in a single layer to 4.70 ± 0.05 eV in multiple layers, becoming more stable as layer thickness increases [45]. According to the modified Ohm’s law, a current will inevitably flow when the Fermi levels of two materials do not align, so when Gr and ReSe_2_ materials come into contact, their Fermi levels must align at thermal equilibrium. The electrons migrate from higher to lower Fermi levels, with holes flowing in the opposite direction. Since the Fermi level of ReSe_2_ is higher than that of Gr, electrons occupy a higher energy level and thus diffuse from the ReSe_2_ side to the Gr side. Due to the transfer of electrons, a charged layer of ionized donors, which arises from the loss of electrons, appears on the surface of ReSe_2_, while a space charge layer forms on the Gr surface due to the accumulation of electrons. Considering the requirement for electrical neutrality, the negative charge on the Gr surface equals in magnitude but opposite in sign to the positive charge on the ReSe_2_ surface. Since the electron concentration in Gr is several orders of magnitude higher than the donor concentration in ReSe_2_, the space charge layer in ReSe_2_ is significantly thicker. Analogous to a p-n junction, the electric field in this space charge region inhibits electron flow from ReSe_2_ to Gr. Thermal equilibrium causes the energy band of ReSe_2_ to bend upward, creating a barrier against electron migration to Gr. The Fermi levels of ReSe_2_ and Gr eventually align at thermal equilibrium, forming a defined space charge region width, stable built-in electric field, and a certain built-in potential difference, as shown in Figure 5c. For electrons flowing from Gr to ReSe_2_, they need to overcome the energy barrier.

Figure 5c illustrates the energy-band structure of the ReSe_2_/Gr heterojunction. Since the Fermi level of ReSe_2_ is higher than that of Gr (as discussed previously in the manuscript), electrons in ReSe_2_ diffuse into Gr upon contact, thereby generating a built-in electric field directed from ReSe_2_ toward Gr. Figure 5d presents the operating mechanism of the heterojunction device. Under illumination, photogenerated electron–hole pairs are created in ReSe_2_, which serves as the primary light-absorbing layer. These photocarriers are effectively separated by the built-in electric field at the ReSe_2_/Gr interface. Meanwhile, due to the presence of hole-trapping defect states on the surface of Gr, the separated photogenerated holes are captured by Gr, significantly reducing the recombination probability of electrons and holes. Furthermore, because Gr is not connected to the external circuit, the accumulated holes produce an upward local electric field, functioning as an effective positive gate voltage. This local photogating effect raises the Fermi level of ReSe_2_, increases the free electron concentration within the ReSe_2_ channel, and enhances the channel conductivity, resulting in a higher photocurrent compared to the pristine ReSe_2_ device.

To highlight the scientific value of our research, we conduct a benchmark comparison of the responsivity, external quantum efficiency (EQE), specific detectivity (D*), and response speed obtained in this study against those of recently published ReSe_2_-based photodetectors, ReSe_2_/graphene (Gr) heterostructured devices, and other 2D material-based photodetectors as summarized in Table 2. These comparisons clearly indicate that our ReSe_2_/Gr device achieves superior photoresponse and quantum efficiency relative to recently reported ReSe_2_-based photodetectors, thereby highlighting the strong scientific relevance and improved optoelectronic performance enabled by integrating graphene with ReSe_2_.

In summary, the role of Gr in the ReSe_2_/Gr heterojunction can be described in two aspects: (1) forming a built-in electric field with ReSe_2_ to facilitate efficient separation of photogenerated electron–hole pairs; (2) capturing photogenerated holes through surface defects to suppress recombination and simultaneously inducing a local positive photogating voltage, thereby increasing the electron concentration in the ReSe_2_ channel.

Based on the high responsivity (294 mA/W), high external quantum efficiency (EQE) (68.75%), and fast response speed (40/62 ms) of the ReSe_2_/Gr heterojunction photodetector, this device can flexibly adapt to practical scenarios such as flexible electronics, biomedical low-light imaging, short-range optical communication, and ambient light monitoring [46,47]. Its simple structure that does not require complex chemical doping is more conducive to integration into micro-sensing systems, providing a practical solution for the development of high-performance portable optoelectronic devices.

## 4. Conclusions

This study presents the enhancement of photodetection performance for the traditional ReSe_2_ photodetector architecture through the incorporation of a Gr auxiliary layer, detailing the fabrication process, materials characterization, and the electrical and optical performance evaluation of the ReSe_2_/Gr and ReSe_2_ phototransistors. The ReSe_2_/Gr phototransistor achieves a high photoresponsivity of 294 mA/W, a D* of 1.74 × 10^11^ Jones, an EQE of 68.75%, and a rapid response speed of 40/62 ms. When compared with conventional ReSe_2_ phototransistor, the ReSe_2_/Gr phototransistor facilitates a significant increase in photoresponsivity (approximately 3.58 times) and external quantum efficiency (approximately 3.59 times). The findings suggest that introduction of Gr auxiliary layer under channel region for 2D phototransistors markedly bolsters the photogating voltage effect, thereby enhancing photosensitivity. This approach provides a valuable method to improve the performance of 2D material-based photodetectors.

## Figures and Tables

**Table 1:** Comparison of key performance parameters between ReSe_2_/Gr device and ReSe_2_ devices.

Device	Channel Size	*R* (mA/W)	*EQE* (%)	*τ*_r_/*τ*_f_ (ms)
ReSe_2_/Gr	L = 19 μm W = 6 μm	294	68.75	40/62
ReSe_2_	L = 4.1 μm W = 18.7 μm	82	19.13	50/67

**Table 2:** Comprehensive comparison of our device with recent ReSe_2_-, ReSe_2_/Gr heterojunction-, and other 2D-material-based optoelectronic sensors.

Photodetector	Responsivity (mA/W)	EQE (%)	D* (Jones)	Rise Time/Fall Time (ms)	Ref.
Bi_2_Se_3_/ReSe_2_	64	/	1.1 × 10^11^	0.345/0.336	[31]
ReSe_2_	43	/	>10^3^	400.6/752.4	[32]
ReSe_2_/PtS_2_	30.9	5.59	1.96 × 10^11^	4/4	[33]
WSe_2_/Ta_2_NiSe_5_	14.6	2.85	8 × 10^9^	4.8/4	[34]
ReSe_2_/Gr	294	68.75	1.74 × 10^11^	40/62	This work

## Data Availability

The data presented in this study are available upon request from the corresponding authors.

## Supplementary Materials

The following supporting information can be downloaded at https://www.mdpi.com/article/10.3390/s26010115/s1, Figure S1: (a) Schematic of ReSe2 FET. (b) AFM image of the ReS2 flake in ReSe2 FET; Figure S2: Absorbance spectrum of ReSe2 as a function of incident light wavelength; Figure S3: (a) The transfer curves of the ReSe2 device under different illumination intensities. (b) The output curves of the ReSe2 device under different illumination intensities; Figure S4: (a) The ReSe2 phototransistor’s response to light pulse. (b) The normalization of a single light pulse cycle showing the rise and fall times.
