# Supplementary material for: Enhancing the Photoresponsivity and External Quantum Efficiency of ReSe2 Photodetectors Through the Insertion of a Graphene Auxiliary Layer
Source: Sensors (Basel). 2025 Dec 24;26(1):115. doi: 10.3390/s26010115 (PMC12787958; doi:10.3390/s26010115)
Supplement: Supplementary file 1 [file sensors-26-00115-s001.zip › sensors-4001749-supplementary.pdf]

Supplementary Materials

Enhancing the Photoresponsivity and External Quantum Efficiency of $ReSe_2$ Photodetectors Through the Insertion of a Graphene Auxiliary Layer

Qiutong Liu, Beiyang Jin, Yutong Li, Peishuo Li, Jingyu Zhang, Yankun Chen, Chenkai Hu and Wei Li*

School of Microelectronics, Northwestern Polytechnical University, 127 Youyi West Road, Xi'an 710072, People's Republic of China

*Corresponding author: weili2019@nwpu.edu.cn

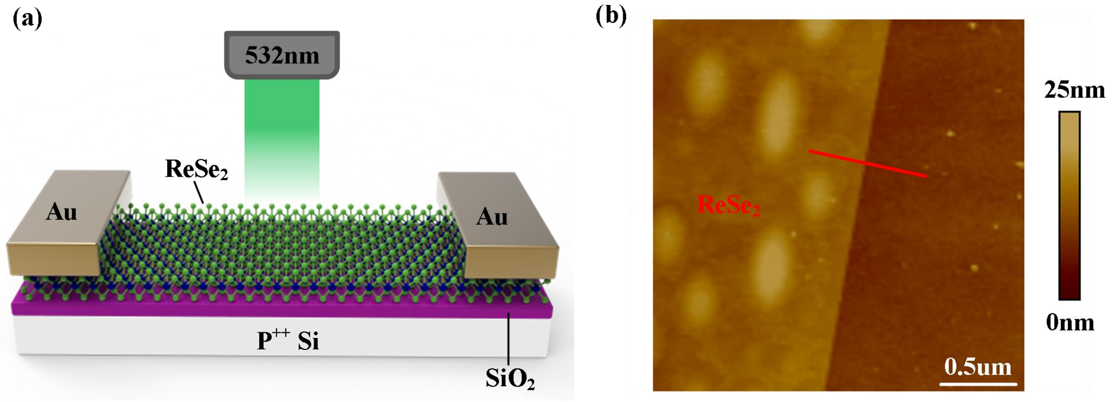

Figure S1. (a) Schematic of $ReSe_2$ FET. (b) AFM image of the ReS_2 flake in $ReSe_2$ FET.

Figure S1(a) is a schematic of the $ReSe_2$ device on a 285 nm SiO_2/Si substrate. The $ReSe_2$ device uses gold (Au) as the source/drain electrodes on both sides of the $ReSe_2$, exposing the middle part of the $ReSe_2$ to 532nm incident light. By using an atomic force microscope (AFM), the thickness of the $ReSe_2$ flake in the $ReSe_2$ device was measured to be about 6.5 nm.

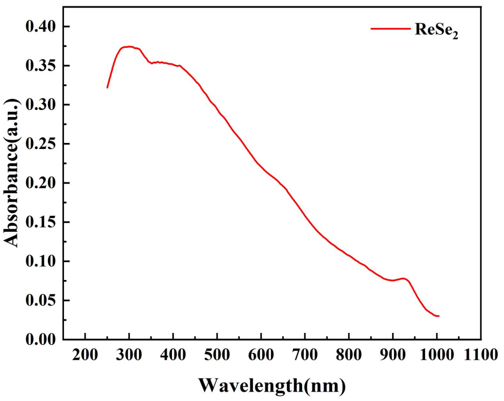

Figure S2. Absorbance spectrum of $ReSe_2$ as a function of incident light wavelength.

Figure S2 shows the absorption coefficient of $ReSe_2$ for light of various wavelengths. As shown in figure, the absorption of 532 nm light by $ReSe_2$ is at a relatively balanced level—the absorption coefficient is neither excessively high nor too low.

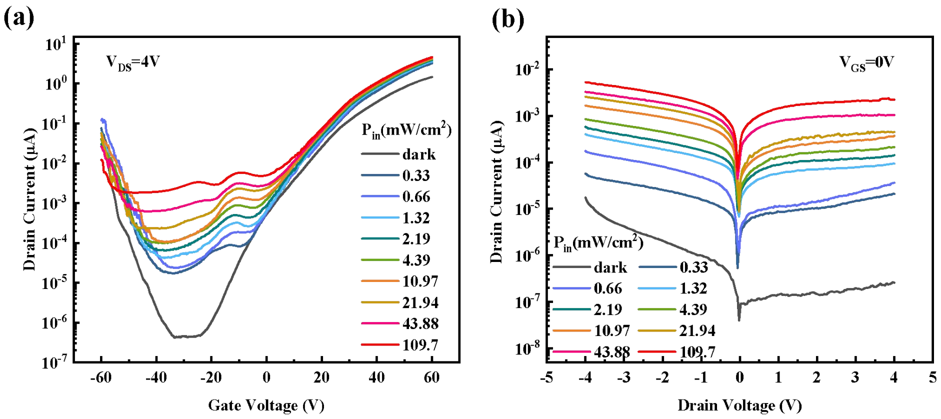

Figure S3. (a) The transfer curves of the $ReSe_2$ device under different illumination intensities. (b) The output curves of the $ReSe_2$ device under different illumination intensities.

Figure S3(a) shows the transfer characteristic curves with V_{GS} from -60V to 60V under different incident light intensities, with the incident light power density increasing from 0.33 mW/cm² to 109.7 mW/cm², and the spot diameter being 30um. **Figure S3(b)** shows the output characteristic curves with V_{DS} from -4V to 4V under different incident light intensities. At an incident light power density of 109.7 mW/cm², the device exhibits a photocurrent of 5.28×10^{-9} A at V_{DS} = -4 V, with a maximum photo-to-dark current ratio of 302, demonstrating an outstanding optical response. With the increase of incident laser power density, a significant increase in the output current can be observed.

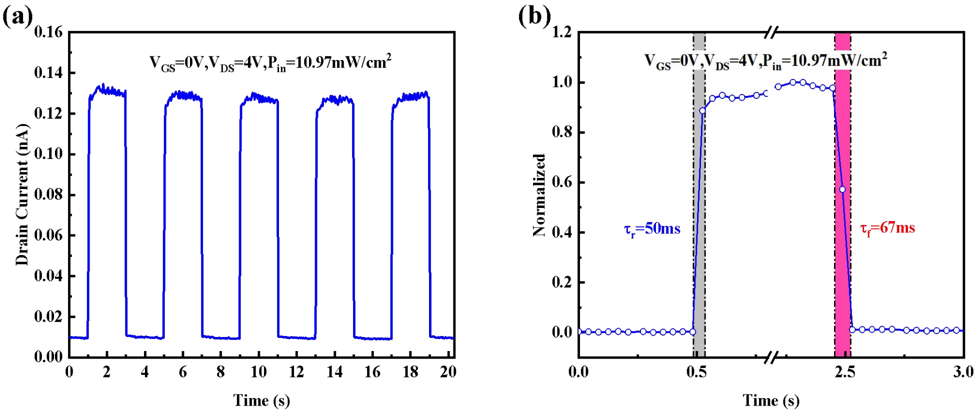

Figure S4. (a) The $ReSe_2$ phototransistor's response to light pulse. (b) The normalization of a single light pulse. cycle showing the rise and fall times.

To comparison with the $ReSe_2$/Gr device, we measured the photo pulse output characteristics of the $ReSe_2$ phototransistor under the conditions of V_{DS} = 4 V and V_{GS} = 0 V. By using a signal generator to control an adjustable light source, a series of measurements were taken by laser pulses with the durations of 2s and intervals of 2s. **Figure S4 (a)** illustrates the response of $ReSe_2$ phototransistor to light pulse with the power density of 10.97 mW/cm^2, showing a rapid increase in drain current upon light exposure and a swift decrease when the light ceases. Further normalization of a single light pulse cycle is shown in **Figure S4 (b)**, we observed a rise time of 40 ms and a fall time of 62 ms, demonstrating the quick response of $ReSe_2$ phototransistor.
